# When Psychosis is Not Psychosis: A Quality Improvement Initiative to Reduce Delirium Misdiagnosis Across Hospital Departments

**DOI:** 10.1192/bjo.2026.11410

**Published:** 2026-06-30

**Authors:** Regina Rachel Khakha, Seema Rani

**Affiliations:** 1 ESIC Medical College and Hospital, Faridabad, India; 2 University College of Medical Sciences, New Delhi, India

## Abstract

**Aims:**

Delirium is frequently misdiagnosed as psychosis in general hospital settings, leading to inappropriate Psychiatry referrals and delays in medical management. This quality improvement initiative aimed to reduce the misdiagnosis of delirium as psychosis by improving early recognition across non-psychiatric departments. Secondary aims were to examine department-wise variation in misdiagnosis, assess uptake of the Confusion Assessment Method for the ICU (CAM-ICU), compare the effectiveness of different CAM-ICU teaching strategies, and evaluate changes in time to delirium management.

**Methods:**

This quality improvement project was conducted in a tertiary care teaching hospital over six months and included referrals from Medicine, Surgery, Obstetrics & Gynaecology, Orthopaedics, and ICU. A baseline audit was undertaken over two months, including all inpatient Psychiatry referrals labelled “suspected psychosis” (n=130). Data collected included referring department, final diagnosis (delirium or psychosis), CAM-ICU use prior to referral, and time to initiation of delirium management. Pre-intervention CAM-ICU use was 0% across all departments.

A targeted educational intervention was delivered over four weeks. Departments received CAM-ICU training using different methods: didactic teaching sessions (Medicine, Surgery), bedside demonstrations (ICU, Orthopaedics), and visual quick-reference materials with brief ward-based teaching (Obstetrics & Gynaecology). A post-intervention re-evaluation was conducted over a further two months (n=126) using identical outcome measures. Department-wise changes in misdiagnosis rates, CAM-ICU uptake, and time to delirium management were analysed and compared across teaching modalities.

**Results:**

At baseline, 82 of 130 referrals (63%) for suspected psychosis were diagnosed as delirium. Misdiagnosis rates varied across departments: Medicine 70%, Surgery 68%, Orthopaedics 62%, Obstetrics & Gynaecology 58%, and ICU 45%. Mean time to initiation of delirium management was 22 hours.

Following the intervention, overall misdiagnosis was reduced to 39 of 126 referrals (31%). Department-wise misdiagnosis rates decreased to Medicine 38%, Surgery 42%, Orthopaedics 25%, Obstetrics & Gynaecology 34%, and ICU 18%. Overall CAM-ICU uptake increased to 56%, with highest use in departments receiving bedside-based training (ICU 72%, Orthopaedics 65%). Mean time to delirium management reduced from 22 hours to 11 hours.

**Conclusion:**

This quality improvement initiative demonstrates that a substantial proportion of referrals labelled as psychosis represent delirium, with marked department-wise variation. Structured CAM-ICU training improved screening uptake, reduced misdiagnosis, and shortened delays to treatment. Bedside and interactive teaching methods were associated with greater improvements than didactic approaches alone. Embedding routine delirium screeninginto inpatient referral pathways is a feasible and effective strategy to enhance patient safety and consultation–liaison psychiatry practice.

